# Evaluating the impacts of non-pharmaceutical interventions on the transmission dynamics of COVID-19 in Canada based on mobile network

**DOI:** 10.1371/journal.pone.0261424

**Published:** 2021-12-29

**Authors:** Ling Xue, Shuanglin Jing, Hao Wang

**Affiliations:** 1 College of Mathematical Sciences, Harbin Engineering University, Harbin, Heilongjiang, China; 2 Department of Mathematical and Statistical Sciences, University of Alberta, Edmonton, Alberta, Canada; Rutgers University/New Brunswick, UNITED STATES

## Abstract

The COVID-19 outbreak has caused two waves and spread to more than 90% of Canada’s provinces since it was first reported more than a year ago. During the COVID-19 epidemic, Canadian provinces have implemented many Non-Pharmaceutical Interventions (NPIs). However, the spread of the COVID-19 epidemic continues due to the complex dynamics of human mobility. We develop a meta-population network model to study the transmission dynamics of COVID-19. The model takes into account the heterogeneity of mitigation strategies in different provinces of Canada, such as the timing of implementing NPIs, the human mobility in retail and recreation, grocery and pharmacy, parks, transit stations, workplaces, and residences due to work and recreation. To determine which activity is most closely related to the dynamics of COVID-19, we use the cross-correlation analysis to find that the positive correlation is the highest between the mobility data of parks and the weekly number of confirmed COVID-19 from February 15 to December 13, 2020. The average effective reproduction numbers in nine Canadian provinces are all greater than one during the time period, and NPIs have little impact on the dynamics of COVID-19 epidemics in Ontario and Saskatchewan. After November 20, 2020, the average infection probability in Alberta became the highest since the start of the COVID-19 epidemic in Canada. We also observe that human activities around residences do not contribute much to the spread of the COVID-19 epidemic. The simulation results indicate that social distancing and constricting human mobility is effective in mitigating COVID-19 transmission in Canada. Our findings can provide guidance for public health authorities in projecting the effectiveness of future NPIs.

## Introduction

Severe acute respiratory syndrome coronavirus 2 (SARS-CoV-2) caused coronavirus disease 2019 (COVID-19). The COVID-19 has been declared as a public health emergency by the World Health Organization on January 30, 2020 [1]. Many countries and regions around the world were affected and some of them have already experienced the second or even the third wave of COVID-19 outbreaks [2]. The ongoing COVID-19 epidemic presents great threats to public health and significant challenges to the development of the global economy. About 1,720,355 confirmed cases and more than 29,000 deaths were reported in Canada as of November 4, 2021 [3].

Due to the highly contagious nature of COVID-19, reducing social contacts and human mobility has been regarded as the key measures to curb the spread of the COVID-19 epidemic [4, 5]. Canadian public health authorities have implemented NPIs, including suspending local public transportation, closing schools and entertainment places, banning public gatherings, encouraging the public to wash their hands and wearing masks, and suggesting other personal preventive actions [6].

Many mathematical models investigated the impacts of human mobility and NPIs on the spread of the COVID-19 epidemic. Most of these studies focused on evaluating the impact of NPIs in China and the United States [7–11]. To evaluate the impacts of human mobility and NPIs on the spread of the COVID-19 epidemic in Canada, we develop a network model coupled with human mobilities in six categories of places, namely, retail and recreation, grocery and pharmacy, parks, transit stations, workplaces, and residences, and the timing and levels of implementing NPIs.

We fit the number of confirmed COVID-19 cases and deaths to the model, and estimate the average effective reproduction numbers, the transmission rate and the probability of a susceptible individual being infected in Alberta (AB), British Columbia (BC), Manitoba (MB), New Brunswick (NB), Newfoundland and Labrador (NL), Nova Scotia (NS), Ontario (ON), Quebec (QC), and Saskatchewan (SK) from February 15 to December 13, 2020.

## Methods

In this section, we introduce the sources of data and present the network model that was developed to evaluate the impact of human mobility and NPIs on the spread of the COVID-19 epidemic in Canada.

### Data collection and analysis

To capture the COVID-19 transmission dynamics in Alberta (AB), British Columbia (BC), Manitoba (MB), New Brunswick (NB), Newfoundland and Labrador (NL), Nova Scotia (NS), Ontario (ON), Quebec (QC), and Saskatchewan (SK), we fit publicly available incidence data of COVID-19 for Canada to the mathematical model incorporating human mobility. We obtain the number of COVID-19 cases in nine Canadian provinces from Johns Hopkins University Center for Systems Science and Engineering [12] (see Fig A.1 of S1 File). The mobility data is obtained from Google COVID-19 Community Mobility Reports (CMR) at https://google.com/covid19/mobility [13] as is plotted in Fig A.2 of S1 File. The areas under study are divided into six different categories, which can be summarised as retail and recreation, grocery and pharmacy, parks, transit stations, workplaces, and residences. CMR show how visits and duration of stay at types of different places change around the baseline. The baseline is the median value for the corresponding day of the week during the five weeks from January 3 to February 6, 2020 [13]. We use the average of the previous seven days to approximate the missing values.

Cross-correlation is a standard method of estimating the correlation between different sets of data (see Appendix B of S1 File). To determine which factor impacts the dynamics of COVID-19 most, we use the cross-correlation analysis [14–18] to derive the correlation between the Google Community Mobility Reports data and the weekly number of confirmed cases from February 15 to December 13, 2020. We also evaluate the impact of the time-lag of mobility data with respect to the incidence data on the COVID-19 outbreak.

**Fig 1:**
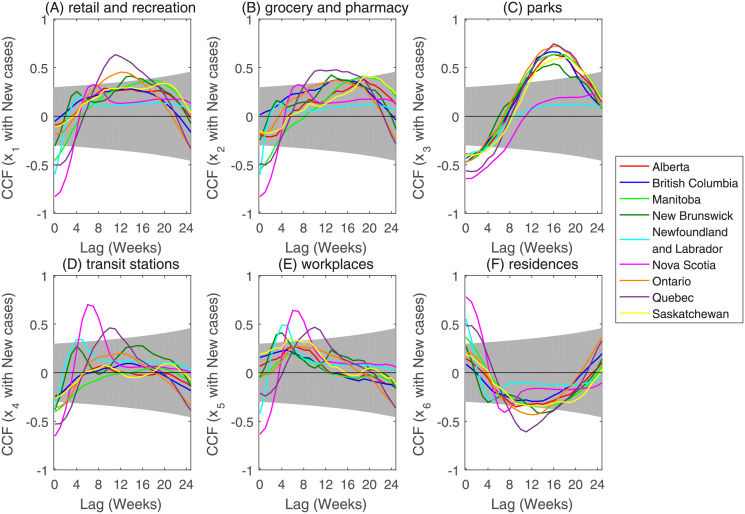
Correlations between Google Community Mobility Reports data and the weekly number of confirmed cases with different lags (in weeks) for each region. The variables *x*_1_(A), *x*_2_(B), *x*_3_(C), *x*_4_(D), *x*_5_(E), and *x*_6_(F) denote retail and recreation, grocery and pharmacy, parks, transit stations, workplaces, and residences. The gray area represents the 95% confidence intervals.

The cross-correlation between the weekly number of confirmed cases and the Google Community Mobility Reports data for the nine Canadian provinces under study are as shown in Fig 1. The correlation between the weekly number of confirmed cases and the Google Community Mobility Reports data is statistically significant, and the cross-correlation between the weekly number of confirmed cases and human mobility data in residential areas are different from that between the weekly number of confirmed cases and human mobility data in other five classes of places (see Fig 1). Specifically, the human mobility in residential areas and the weekly number of confirmed cases are negatively correlated when the average time-lag in each province is four weeks, while the human mobility in the other five classes of places and the weekly number of confirmed cases are positively correlated when the average time-lag in each province was four weeks. The above results show that activities around the residences do not accelerate the spread of the COVID-19 epidemic. The average time-lag between human mobility in parks (eight weeks) and the weekly number of confirmed cases is longer than that between human mobility in retail and recreation (four weeks), grocery and pharmacy (six weeks), transit stations (six weeks), and workplaces (one week) during the presence of positive correlation. The cross-correlation analysis between Google Community Mobility Reports data and the weekly number of confirmed death cases are shown in Fig A.3 of S1 File.

### Model formulation

**Fig 2:**
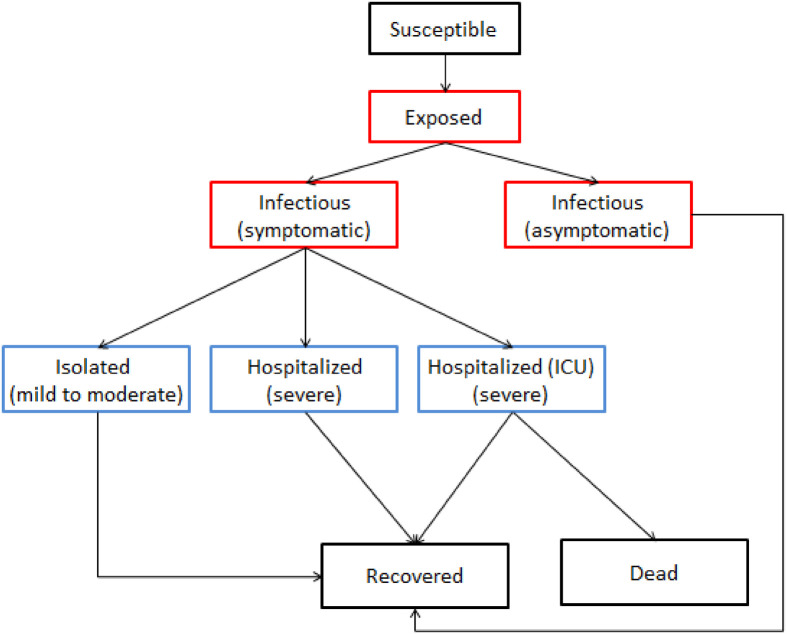
Schematic diagram of the mathematical model.

In order to mimic the spread of the COVID-19 epidemic, we construct a disease transmission model with a population mobile network. The whole population is divided into nine classes, namely, *S*(*t*), *E*(*t*), *A*(*t*), *I*(*t*), *Q*(*t*), *H*(*t*), *H*_*i*_(*t*), *R*(*t*), and *D*(*t*) that represent the numbers of individuals who were: (i) susceptible; (ii) exposed; (iii) infectious (asymptomatic); (iv) infectious (symptomatic); (v) isolated symptomatic and infected (mild to moderate); (vi) hospitalized (severe) not requiring ICU care; (vii) hospitalized (severe) requiring ICU care; (viii) recovered; and (ix) who had died, respectively. The total population at time *t* is denoted by *N*(*t*). The population flow among those compartments is shown in Fig 2.

The population mobile network consisted of nodes representing six categories of places, namely, retail and recreation, grocery and pharmacy, parks, transit stations, workplaces, and residences. Since the human mobility of individuals in each place is different during the day and night [19], we develop different models for the daytime and the night. During the daytime, the human mobility mainly occurs in retail and recreation, grocery and pharmacy, parks, transit stations, workplaces, and residences. During the night, people usually stay in residences.

The model for the daytime is

$$\begin{array}{c} \left\{ \begin{array}{cc} & \frac{\text{d}S}{\text{d}t}=-\frac{S}{N}\sum_{j=1}^{k}\lambda_{j}\left(t\right)N_{j}\left(t\right),\\ & \frac{\text{d}E}{\text{d}t}=\frac{S}{N}\sum_{j=1}^{k}\lambda_{j}\left(t\right)N_{j}\left(t\right)-\sigma E,\\ & \frac{\text{d}A}{\text{d}t}=\rho \sigma E-\gamma_{A}A,\\ & \frac{\text{d}I}{\text{d}t}=\left(1-\rho \right)\sigma E-\epsilon I,\\ & \frac{\text{d}Q}{\text{d}t}=\left(1-\alpha \right)\epsilon I-\gamma_{Q}Q,\\ & \frac{\text{d}H}{\text{d}t}=\alpha P_{QH}\epsilon I-\gamma_{H}H,\\ & \frac{\text{d}H_{i}}{\text{d}t}=\alpha \left(1-P_{QH}\right)\epsilon I-\gamma_{Hi}P_{HR}H_{i}-d_{H}\left(1-P_{HR}\right)H_{i},\\ & \frac{\text{d}R}{\text{d}t}=\gamma_{A}A+\gamma_{Q}Q+\gamma_{H}H+\gamma_{Hi}P_{HR}H_{i},\\ & \frac{\text{d}D}{\text{d}t}=d_{H}\left(1-P_{HR}\right)H_{i}. \end{array}\right. \end{array}$$ (1)

For Models (1) and (2), *S*(0), *E*(0), *A*(0), *I*(0), *Q*(0), *H*(0), *H*_*i*_(0), *R*(0), and *D*(0) represent the initial numbers of individuals falling into the respective eight categories listed above. The description of other parameters is shown in Table 1. Here, λ_*j*_(*t*) is the rate of infection in place *j* at time *t*, where *N*_*j*_(*t*)(*j* = 1, 2, 3, 4, 5, 6) represents the number of people in retail and recreation, grocery and pharmacy, parks, transit stations, workplaces, and residences at time *t*, respectively. Therefore, the rate of infection in places *j* at time *t* can be expressed as

$$\begin{array}{c} \lambda_{j}\left(t\right)=\beta_{j}\left(t\right)(\frac{I_{j}}{N_{j}}+\theta_{j}\frac{A_{j}}{N_{j}}+\delta_{j}\frac{E_{j}}{N_{j}}), \end{array}$$

where $I_{j}=\frac{I}{N}N_{j},\;A_{j}=\frac{A}{N}N_{j},\;E_{j}=\frac{E}{N}N_{j}.$ Thus, the rate of infection in places *j* at time *t* can be rewritten as

$$\begin{array}{c} \lambda_{j}\left(t\right)=\beta_{j}\left(t\right)(\frac{I}{N}+\theta_{j}\frac{A}{N}+\delta_{j}\frac{E}{N}). \end{array}$$

**Table 1:** The parameter description of the model.

Parameters	Description (Units)	Value	Source
1/*σ*	The mean length of incubation period (day)	5.2	[20]
*θ*_*j*_	The factor for reduced transmission probability of asymptomatic infected individuals (dimensionless)	0.55	[21, 22]
*δ*_*j*_	The factor for reduced transmission probability of exposed individuals (dimensionless)	0.55	[21, 22]
*ρ*	The proportion of asymptomatic infected individuals (dimensionless)	60%	[23]
1/*ϵ*	Average time to case detection (day)	6	[24]
1/*γ*_*A*_	The asymptomatic infectious period (day)	8	[25]
1/*γ*_*H*_	Average length of stay in hospitals (not ICU) (day)	10	[26]
1/*γ*_*Hi*_	Average length of ICU stay (day)	21	[27]
1/*γ*_*Q*_	Average time of being isolated (day)	14	[24]
1/*d*_*H*_	Average time from being hospitalized to deaths (day)	21	[27]
1 − *α*	Probability of home isolation (dimensionless)	84.3%	[24, 28]
1 − *P*_*QH*_	Probability of ICU admission (dimensionless)	10%	[29, 30]
1 − *P*_*HR*_	Probability of deaths in ICU (dimensionless)	Table C.1 of S1 File	MCMC
*β*_*j*_(*t*)	Transmission rate (*person* *day*^−1^)	Table C.1 of S1 File	MCMC

**Fig 3:**
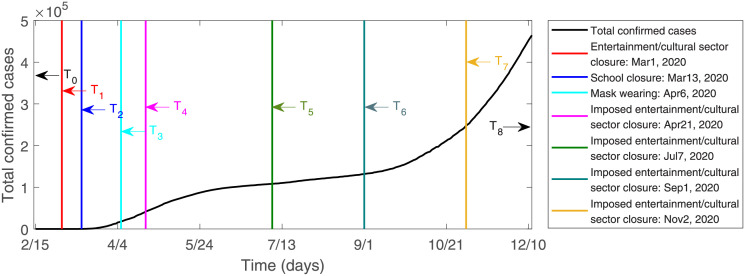
The period when NPIs are implemented in Canada [6]. *T*_1_ (Mar 1, 2020): Entertainment/cultural sector closure. *T*_2_ (Mar 13, 2020): School closure. *T*_3_ (Apr 6, 2020): Mask wearing. *T*_4_ (Apr 21, 2020): Imposed entertainment/cultural sector closure. *T*_5_ (Jul 7, 2020): Imposed entertainment/cultural sector closure. *T*_6_ (Sep 1, 2020): Imposed entertainment/cultural sector closure. *T*_7_ (Nov 2, 2020): Imposed entertainment/cultural sector closure.

During the COVID-19 epidemic, the NPIs taken by the Canadian public health authorities [6] are shown in Fig 3. The specific NPIs are as follows: *T*_1_ (Mar 1, 2020): Entertainment/cultural sector closure. *T*_2_ (Mar 13, 2020): School closure. *T*_3_ (Apr 6, 2020): Mask wearing. *T*_4_ (Apr 21, 2020): Imposed entertainment/cultural sector closure. *T*_5_ (Jul 7, 2020): Imposed entertainment/cultural sector closure. *T*_6_ (Sep 1, 2020): Imposed entertainment/cultural sector closure. *T*_7_ (Nov 2, 2020): Imposed entertainment/cultural sector closure. Therefore, the transmission rate can be expressed as the piecewise function

$$\begin{array}{c} \beta_{j}\left(t\right)=\{\begin{array}{cc} & a_{j1},\;t\in \left[T_{0},\;T_{1}\right),\\ & a_{j2},\;t\in \left[T_{1},\;T_{2}\right),\\ & a_{j3},\;t\in \left[T_{2},\;T_{3}\right),\\ & a_{j4},\;t\in \left[T_{3},\;T_{4}\right),\\ & a_{j5},\;t\in \left[T_{4},\;T_{5}\right),\\ & a_{j6},\;t\in \left[T_{5},\;T_{6}\right),\\ & a_{j7},\;t\in \left[T_{6},\;T_{7}\right),\\ & a_{j8},\;t\in \left[T_{7},\;T_{8}\right]. \end{array} \end{array}$$

The effective reproduction number *R*_*ej*_(*t*) in places *j* at time *t* is

$$\begin{array}{c} R_{ej}\left(t\right)=\frac{\delta_{j}\beta_{j}\left(t\right)}{\sigma }+\frac{\rho \theta_{j}\beta_{j}\left(t\right)}{\gamma_{A}}+\frac{\left(1-\rho \right)\beta_{j}\left(t\right)}{\epsilon }. \end{array}$$

The average effective reproduction number *R*_*e*_(*t*) at time *t* is

$$\begin{array}{c} R_{e}\left(t\right)=\frac{\sum_{j=1}^{k}\delta_{j}\beta_{j}\left(t\right)}{k\sigma }+\frac{\sum_{j=1}^{k}\rho \theta_{j}\beta_{j}\left(t\right)}{k\gamma_{A}}+\frac{\sum_{j=1}^{k}\left(1-\rho \right)\beta_{j}\left(t\right)}{k\epsilon }, \end{array}$$

where the three terms represent the numbers of new cases generated by exposed, asymptomatic, and symptomatic individuals, respectively.

The probability of a susceptible individual being infected in places *j* at time *t* is defined as

$$\begin{array}{c} IP_{j}\left(t\right)=\beta_{j}\left(t\right)(\frac{I}{N}+\theta_{j}\frac{A}{N}+\delta_{j}\frac{E}{N}). \end{array}$$

Similarly, the model for nighttime is

$$\begin{array}{c} \left\{ \begin{array}{cc} & \frac{\text{d}S}{\text{d}t}=-\beta_{6}\left(t\right)S(\frac{I}{N}+\theta_{6}\frac{A}{N}+\delta_{6}\frac{E}{N}),\\ & \frac{\text{d}E}{\text{d}t}=\beta_{6}\left(t\right)S(\frac{I}{N}+\theta_{6}\frac{A}{N}+\delta_{6}\frac{E}{N})-\sigma E,\\ & \frac{\text{d}A}{\text{d}t}=\rho \sigma E-\gamma_{A}A,\\ & \frac{\text{d}I}{\text{d}t}=\left(1-\rho \right)\sigma E-\epsilon I,\\ & \frac{\text{d}Q}{\text{d}t}=\left(1-\alpha \right)\epsilon I-\gamma_{Q}Q,\\ & \frac{\text{d}H}{\text{d}t}=\alpha P_{QH}\epsilon I-\gamma_{H}H,\\ & \frac{\text{d}H_{i}}{\text{d}t}=\alpha \left(1-P_{QH}\right)\epsilon I-\gamma_{Hi}P_{HR}H_{i}-d_{H}\left(1-P_{HR}\right)H_{i},\\ & \frac{\text{d}R}{\text{d}t}=\gamma_{A}A+\gamma_{Q}Q+\gamma_{H}H+\gamma_{Hi}P_{HR}H_{i},\\ & \frac{\text{d}D}{\text{d}t}=d_{H}\left(1-P_{HR}\right)H_{i}. \end{array}\right. \end{array}$$ (2)

### Parameter estimation

We fit Models (1) and (2) to the weekly number of confirmed cases to quantify the dynamics of COVID-19 in nine Canadian provinces. We simulate the epidemic for each province from February 15, 2020. Since daily numbers of new cases were not reported in Canada on February 15, 2020, we assume that the initially daily number of newly infected individuals on February 15, 2020 is zero in simulations. The size of total population and the age pyramid for the nine provinces are obtained from Statistics Canada [31]. In the simulations, we assume that the population is spatially uniformly distributed before February 15, 2020. After February 15, 2020, we use the Google Community Mobility Reports data to quantify the number of people in six types of places (retail and recreation, grocery and pharmacy, parks, transit stations, workplaces, and residences). Since the incubation period of COVID-19 is around 5.2 days [20], the incubation rate *σ* = 1/5.2. Regarding the survival rate and length of ICU stay duo to COVID-19 infection, we assume that the average length of ICU stay and the average time from hospitalization to deaths are both 21 days, and the average length of hospital stay rather than ICU is ten days, and the duration of quarantine for exposed cases is 14 days [27], that is, 1/*γ*_*Hi*_ = 1/*d*_*H*_ = 21, 1/*γ*_*H*_ = 10, and 1/*γ*_*Q*_ = 14. Moreover, we assume that the asymptomatic infectious period is eight days [25]. Thus, we let *γ*_*A*_ = 1/8 per day. We assume that the average time to case detection is six days [24], that is, 1/*ϵ* = 6. Around 30% − 60% of people infected with due to COVID-19 are asymptomatic or only have mild symptoms, and the transmissibility is lower, but still significant [23]. Thus, we assume that the probability of asymptomatic infected individuals is *ρ* = 60%. We set *θ*_*j*_ = *δ*_*j*_ = 0.55 [21, 22] by assuming that exposed and asymptomatic infected individuals have lower transmissibility. We additionally assume that the probability of home isolation is 84.3% and the probability of ICU admission is 10% [24, 28–30], that is, 1 − *α* = 84.3% and 1 − *P*_*QH*_ = 10%.

In order to simulate the number of new cases per week in nine Canadian provinces, the feasibility of the model is verified by the actual number of newly infected cases. Next, we use the MCMC method [32, 33] for 50000 iterations with a burn-in of 45000 iterations to fit the Models (1) and (2). We estimate the unknown parameters and initial conditions for the Models (1) and (2) ($\hat{\chi }=\left(E\left(0\right),a_{ji}\left(j=1,2,\cdots ,6,\;i=1,2,\cdots ,8\right),P_{HR}\right)$), using the weekly number of confirmed cases in Canada. Let $C(t,\hat{\chi })$ represents the cumulative number of confirmed cases. The dynamics of $C(t,\hat{\chi })$ is

$$\begin{array}{c} \frac{\text{d}C(t,\hat{\chi })}{\text{d}t}=\epsilon I. \end{array}$$

The weekly number of confirmed cases is

$$\begin{array}{c} P_{C}\left(i,\hat{\chi }\right)=\int_{\text{week}\;i}\epsilon I\;\text{d}t, \end{array}$$ (3)

where *P*_*C*_ represents the weekly number of confirmed cases, and the time step is hours in the simulations, then *C*(0) = *P*_*C*_(0). We have Ψ independent observations from the data, representing the number of confirmed cases on the *i*-th week, where *i* = 1, …, Ψ. Let *ε* be the fitting error, and *ε* follows the additive independent Gaussian distribution with mean zero and unknown variance *ξ*^2^. Thus, the observations ${\bar{Y}}_{C}$ can be expressed as follows

$$\begin{array}{c} {\bar{Y}}_{C_{i}}=P_{C}\left(i,\hat{\chi }\right)+\varepsilon ,\;\;\varepsilon ∼N\left(0,I\xi^{2}\right). \end{array}$$ (4)

For simplicity, we assume that the unknown parameters $\hat{\chi }$ of system is an independent Gaussian prior specification. Therefore,

$$\begin{array}{c} {\hat{\chi }}_{j}∼N\left(\nu_{j},\varphi_{j}^{2}\right),\;\;j=1,\ldots ,M. \end{array}$$

where *M* is the number of unknown parameters. We further assume that the inverse of the error variance follows a gamma distribution as prior with the form

$$\begin{array}{c} p\left(\xi^{-2}\right)∼\Gamma (\frac{n_{0}}{2},\frac{n_{0}}{2}S_{0}^{2}), \end{array}$$

where $S_{0}^{2}$ and *n*_0_ are the prior mean and prior accuracy of variance *ξ*^2^, respectively.

The likelihood function $p({\bar{Y}}_{C}|\hat{\chi },\xi^{2})$ for Ψ independent identically distributed observations from Eq (4) with a Gaussian error model is

$$\begin{array}{c} p\left({\bar{Y}}_{C}|\hat{\chi },\xi^{2}\right)=(\frac{1}{\sqrt{2\pi \xi^{2}}}{)}^{\Psi }\text{exp}[\frac{-\text{SS}(\hat{\chi })}{2\xi^{2}}], \end{array}$$

where $\text{SS}(\hat{\chi })$ represents the sum of squares function

$$\begin{array}{c} \text{SS}\left(\hat{\chi }\right)=\overset{\Psi }{\sum_{i=1}}[{\left({\bar{Y}}_{C_{i}}-P_{C}\left(i,\hat{\chi }\right)\right)}^{2}]. \end{array}$$

The conditional distribution $p(\xi^{-2}|{\bar{Y}}_{C},\hat{\chi })$ can be expressed as follows

$$\begin{array}{c} \begin{array}{cc} p(\xi^{-2}|{\bar{Y}}_{C},\hat{\chi }) & \propto p\left({\bar{Y}}_{C}|\xi^{-2},\hat{\chi }\right)p\left(\xi^{-2}\right)\\ & ={\left(\frac{1}{\sqrt{2\pi }\xi^{-1}}\right)}^{\Psi }\text{exp}[\frac{-\text{SS}(\hat{\chi })}{2\xi^{-2}}]\frac{{\left(\frac{n_{0}}{2}S_{0}^{2}\right)}^{\frac{n_{0}}{2}}}{\Gamma (\frac{n_{0}}{2})}(\xi^{-2}{)}^{-\frac{n_{0}}{2}-1}\text{exp}[-\frac{n_{0}S_{0}^{2}}{2\xi^{-2}}]\\ & \propto (\xi^{-2}{)}^{-\frac{\Psi +n_{0}}{2}-1}\text{exp}[-\frac{\text{SS}\left(\hat{\chi }\right)+n_{0}S_{0}^{2}}{2\xi^{-2}}]. \end{array} \end{array}$$

According to the conditional conjugacy property of the Gamma distribution [34, 35], the conditional distribution $p(\xi^{-2}|{\bar{Y}}_{C},\hat{\chi })$ is also a Gamma distribution with

$$\begin{array}{c} p\left(\xi^{-2}|{\bar{Y}}_{C},\hat{\chi }\right)=\Gamma (\frac{\Psi +n_{0}}{2},\frac{\text{SS}\left(\hat{\chi }\right)+n_{0}S_{0}^{2}}{2}), \end{array}$$

based on which sample and update *ξ*^−2^ for other parameters within each run of Metropolis Hastings simulation steps. Since we assume independent Gaussian prior specification for parameters $\hat{\chi }$, the prior sum of squares for the given parameters $\hat{\chi }$ can be calculated as follows

$$\begin{array}{c} {\text{SS}}_{\text{pri}}\left(\hat{\chi }\right)=\overset{M}{\sum_{i=1}}[\frac{{\hat{\chi }}_{i}-\nu_{i}}{\varphi_{i}}{]}^{2}. \end{array}$$

Hence, for a fixed value of variance *ξ*^2^, the posterior distribution of parameters $\hat{\chi }$ can be expressed as follows

$$\begin{array}{c} \begin{array}{cc} p(\hat{\chi }|{\bar{Y}}_{C},\xi^{2})\propto & p\left({\bar{Y}}_{C}|\hat{\chi },\xi^{2}\right)p\left({\hat{\chi }}_{1}\right)p\left({\hat{\chi }}_{2}\right)\cdots p\left({\hat{\chi }}_{M}\right)=p\left({\bar{Y}}_{C}|\hat{\chi },\xi^{2}\right)\prod_{i=1}^{M}p\left({\hat{\chi }}_{i}\right)\\ = & \left(\frac{1}{\sqrt{2\pi \xi^{2}}}{)}^{\Psi }\text{exp}[\frac{-\text{SS}(\hat{\chi })}{2\xi^{2}}]\prod_{j=1}^{M}\frac{1}{\sqrt{2\pi }\varphi_{j}}\text{exp}[-\frac{{\left({\hat{\chi }}_{j}-\nu_{j}\right)}^{2}}{2\varphi_{j}^{2}}\right]\\ = & \left(\frac{1}{\sqrt{2\pi \xi^{2}}}{)}^{\Psi }\text{exp}[\frac{-\text{SS}(\hat{\chi })}{2\xi^{2}}](\frac{1}{\sqrt{2\pi }}{)}^{M}\frac{1}{\varphi_{1}\varphi_{2}\cdots \varphi_{M}}\text{exp}[-\frac{1}{2}\overset{M}{\sum_{j=1}}(\frac{{\hat{\chi }}_{j}-\nu_{j}}{\varphi_{j}}{)}^{2}\right]\\ \propto & \text{exp}[-\frac{1}{2}(\frac{\text{SS}(\hat{\chi })}{\xi^{2}}+{\text{SS}}_{\text{pri}}\left(\hat{\chi }\right))], \end{array} \end{array}$$

and the posterior ratio needed in the Metropolis-Hastings acceptance probability can be written as follows

$$\begin{array}{c} \frac{p(\hat{\chi^{1}}|{\bar{Y}}_{C},\xi^{2})}{p(\hat{\chi^{2}}|{\bar{Y}}_{C},\xi^{2})}=\text{exp}[-\frac{1}{2}(\frac{\text{SS}(\hat{\chi^{2}})}{\xi^{2}}-\frac{\text{SS}(\hat{\chi^{1}})}{\xi^{2}})+\frac{1}{2}({\text{SS}}_{\text{pri}}\left(\hat{\chi^{2}}\right)-{\text{SS}}_{\text{pri}}\left(\hat{\chi^{1}}\right))]. \end{array}$$

Accordingly, the new unknown parameter value $\hat{\chi^{2}}$ will be accepted with probability

$$\begin{array}{c} \text{min}(1,\;\frac{p(\hat{\chi^{1}}|{\bar{Y}}_{C},\xi^{2})}{p(\hat{\chi^{2}}|{\bar{Y}}_{C},\xi^{2})}). \end{array}$$

We find the local minimum of $\text{SS}(\hat{\chi })$ using nonlinear least square approach to obtain the estimated value of each sample. The minimum value of these estimated $\hat{\chi }$ is used as the initial guess in MCMC simulation. Prior information of unknown parameters is given by *E*(0)∈(0, 1000), *a*_*ji*_ ∈ (0, 10)(*j* = 1, 2, ⋯, 6, *i* = 1, 2, ⋯, 8), *P*_*HR*_ ∈ (0, 1), and the proposal density follows a multivariate normal distribution.

**Fig 4:**
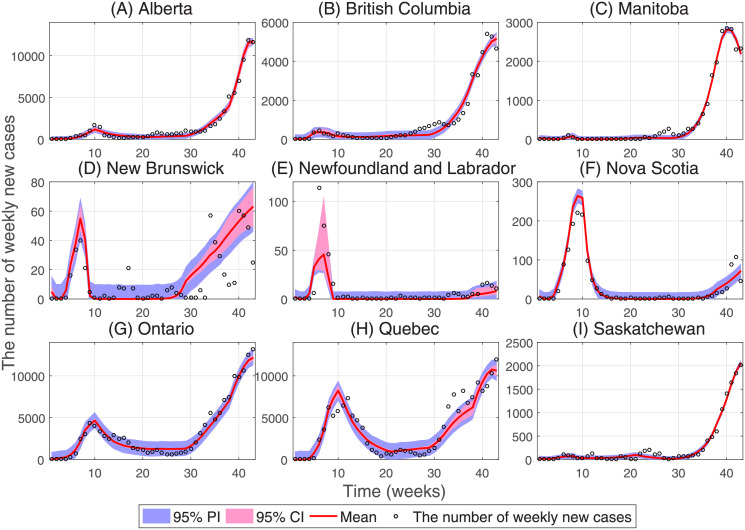
Curve fitting of Models (1) and (2) to weeky total number of confirmed cases for nine provinces of Canada. The dots indicate the number of total confirmed new cases each week. The red curve represents the number of estimated cases, and the 95% confidence and prediction intervals are shown as light red and light blue, respectively.

**Fig 5:**
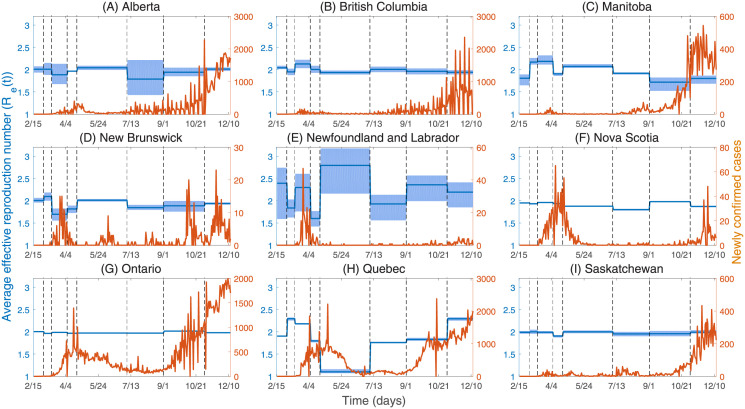
The average effective reproduction numbers (*R*_*e*_(*t*)) and the number of newly confirmed cases. The black dotted line indicates the time when NPIs are implemented as follows. *T*_1_ (Mar 1, 2020): Entertainment/cultural sector closure. *T*_2_ (Mar 13, 2020): School closure. *T*_3_ (Apr 6, 2020): Mask wearing. *T*_4_ (Apr 21, 2020): Imposed entertainment/cultural sector closure. *T*_5_ (Jul 7, 2020): Imposed entertainment/cultural sector closure. *T*_6_ (Sep 1, 2020): Imposed entertainment/cultural sector closure. *T*_7_ (Nov 2, 2020): Imposed entertainment/cultural sector closure.

By fitting Models (1) and (2) to the time series of new cases reported in nine Canadian provinces as shown in Fig 4, we plotted the average effective reproduction number (*R*_*e*_(*t*)) as shown in Fig 5. The mean and standard deviation of the parameters and model initial values are shown in Tables C.1 and C.2 of S1 File.

## Results

In this section, we calculated the effective reproduction numbers, transmission rates, infection probabilities, and quantified the impact of human mobility on COVID-19 in nine Canadian provinces.

### Average effective reproduction numbers in nine Canadian provinces

Fig 5 shows that the average effective reproduction numbers in nine Canadian provinces are all greater than one from February 15, 2020 to December 13, 2020, which indicates that the COVID-19 epidemic has not been under control. In particular, the effective reproduction numbers for Alberta, British Columbia, Manitoba, New Brunswick, Nova Scotia, Ontario, and Saskatchewan have changed very little from February 15, 2020 to December 13, 2020. However, for Newfoundland and Labrador, and Quebec, the effective reproduction numbers have changed dramatically in different time periods.

### The impact of mobility on COVID-19

Mobility in Canada dropped sharply in March 2020: e.g., in March and April, 2020, the average percentage of retail and recreation visits changed from the baseline by -37.5%, the average percentage of grocery and pharmacy visits changed from the baseline by -12.6%, the average percentage of park visits changed from the baseline by -2.3%, the average percentage of transit station visits changed from the baseline by -49.0%, the average percentage of workplace visits changed from the baseline by -39.8%, and the average percentage of human mobility in residential areas changed from the baseline by 15.8%. However, after April, 2020, the number of people going to the park increased significantly. From May to November, the average percentage of park visits increased by 100.1% from the baseline.

**Fig 6:**
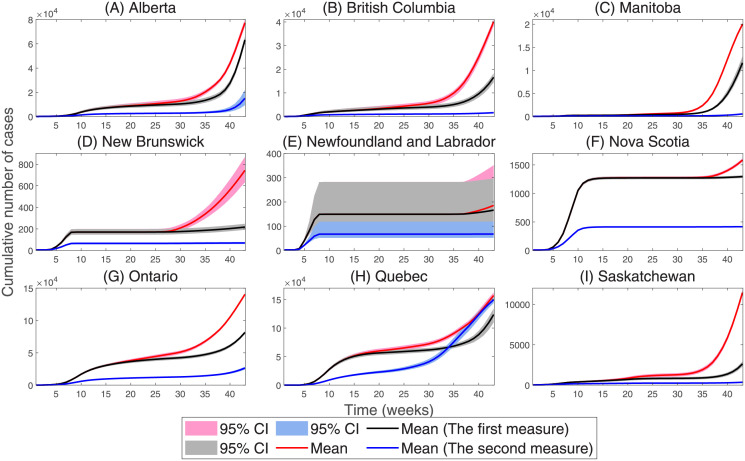
The impact of human mobility on COVID-19. The first measure represents the proportion of people who visited parks after April 25, 2020 as the baseline, and the second measure represents a 20% reduction in the number of population movements.

In this regard, we simulate the spread of COVID-19 when the average percentage of park visits changed from the baseline after April 25, 2020 (see Fig 6). We observe that when the average percentage of park visits changed from the baseline after April 25, 2020, the cumulative number of cases in Canada as of December 13, 2020 is reduced by 32.97%. More predictions for the impact of reducing park visits on the cumulative number of cases in nine Canadian provinces are summarized in Table 2. In addition, we simulate the scenario when the movements of humans are reduced by 20%, as of December 13, 2020, the cumulative number of cases in Canada is reduced by 56.72%. More predictions for the impact of reducing population movement on the cumulative number of cases in nine Canadian provinces are summarized in Table 2.

**Table 2:** The number of cumulative cases varying with population movement.

Province/State	The first measure	The second measure
Alberta	-18.17%	-80.55%
British Columbia	-58.60%	-95.90%
Manitoba	-41.86%	-97.15%
New Brunswick	-70.77%	-90.64%
Newfoundland and Labrador	-10.52%	-63.67%
Nova Scotia	-18.24%	-73.69%
Ontario	-42.02%	-81.18%
Quebec	-21.28%	-4.71%
Saskatchewan	-77.03%	-96.90%

The first measure represents the proportion of people who visited parks after April 25, 2020 as the baseline, and the second measure represents a 20% reduction in the number of population movements.

### The transmission rate in types of different places

**Fig 7:**
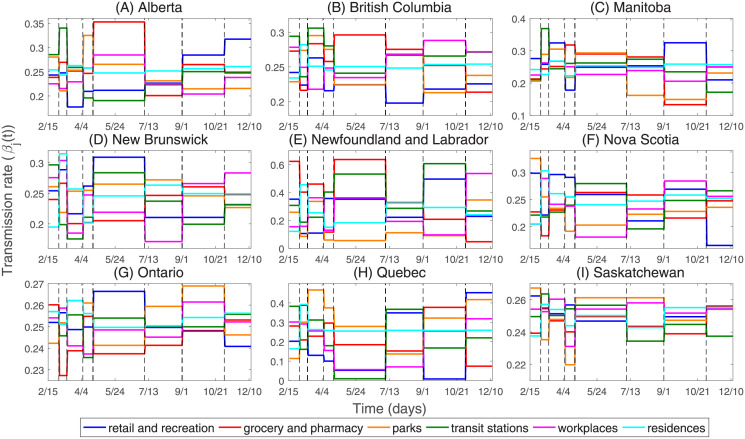
The transmission rates in retail and recreation, grocery and pharmacy, parks, transit stations, workplaces, and residences in nine Canadian provinces. The black dotted line indicates the time when NPIs are implemented as follows. *T*_1_ (Mar 1, 2020): Entertainment/cultural sector closure. *T*_2_ (Mar 13, 2020): School closure. *T*_3_ (Apr 6, 2020): Mask wearing. *T*_4_ (Apr 21, 2020): Imposed entertainment/cultural sector closure. *T*_5_ (Jul 7, 2020): Imposed entertainment/cultural sector closure. *T*_6_ (Sep 1, 2020): Imposed entertainment/cultural sector closure. *T*_7_ (Nov 2, 2020): Imposed entertainment/cultural sector closure.

In order to study the spread of the COVID-19 epidemic in Canada, we plot the transmission rates in types of different places of the nine Canadian provinces from February 15, 2020 to December 13, 2020, as shown in Fig 7. Table 3 shows that the transmission rate changes in different phases are mostly between -10% and 10% in Ontario and Saskatchewan, which shows that the effect of NPIs is not obvious in these two province. However, in Alberta, British Columbia, Manitoba, New Brunswick, Newfoundland and Labrador, Nova Scotia, and Quebec, the transmission rates have changed dramatically at different phases.

**Table 3:** The change of transmission rates in different categories of places under NPIs.

Province/State	Places	*T*_1_	*T*_2_	*T*_3_	*T*_4_	*T*_5_	*T*_6_	*T*_7_
AB	retail and recreation	⇔	↑	↓	⇔	⇔	↓	↓
	grocery and pharmacy	↓	⇔	⇔	↓	↑	↓	⇔
	parks	↑	↓	↓	↑	↑	⇔	⇔
	transit stations	↓	↑	↑	⇔	↓	↓	⇔
	workplaces	⇔	⇔	↓	↓	↑	⇔	↓
	residences	⇔	⇔	⇔	⇔	⇔	⇔	⇔
BC	retail and recreation	⇔	↓	↑	⇔	↑	↓	⇔
	grocery and pharmacy	↑	↓	⇔	↓	⇔	⇔	↑
	parks	↓	⇔	⇔	↑	↓	↑	↓
	transit stations	↑	↓	⇔	↑	↓	⇔	⇔
	workplaces	↑	↑	↓	⇔	↓	⇔	⇔
	residences	↓	↑	⇔	⇔	⇔	⇔	⇔
MB	retail and recreation	⇔	↓	↑	↓	⇔	↓	↑
	grocery and pharmacy	↓	⇔	↓	⇔	⇔	↑	↓
	parks	↓	⇔	↑	↓	↑	⇔	↓
	transit stations	↓	↑	⇔	⇔	⇔	↑	↑
	workplaces	↓	⇔	⇔	⇔	⇔	↑	↓
	residences	⇔	↓	↑	↓	⇔	⇔	⇔
NB	retail and recreation	↓	↑	↓	↓	↑	⇔	⇔
	grocery and pharmacy	↓	↑	⇔	⇔	↓	⇔	⇔
	parks	↑	↓	⇔	⇔	⇔	⇔	⇔
	transit stations	↑	↑	↓	↓	↑	↑	↓
	workplaces	↓	↑	↓	↑	↑	↓	⇔
	residences	↓	↑	↑	↓	⇔	⇔	⇔
NL	retail and recreation	↑	⇔	↓	⇔	↑	↓	↑
	grocery and pharmacy	↑	↓	↑	↓	↑	↑	↑
	parks	↑	↓	↑	⇔	↓	↑	↓
	transit stations	↑	↓	↓	↓	↑	↓	↑
	workplaces	⇔	↓	↑	↓	↑	↑	↓
	residences	↓	↑	↑	↓	↓	⇔	↑
NS	retail and recreation	↑	↓	⇔	↑	↑	↓	↑
	grocery and pharmacy	↑	↓	↓	⇔	⇔	↑	↓
	parks	↑	⇔	↑	⇔	⇔	⇔	⇔
	transit stations	⇔	⇔	⇔	↓	↑	↓	⇔
	workplaces	↓	↑	⇔	↑	↓	↓	⇔
	residences	↓	↑	⇔	⇔	⇔	⇔	⇔
ON	retail and recreation	⇔	⇔	⇔	⇔	⇔	⇔	⇔
	grocery and pharmacy	↑	⇔	⇔	⇔	⇔	⇔	⇔
	parks	⇔	⇔	⇔	⇔	⇔	⇔	⇔
	transit stations	⇔	⇔	⇔	⇔	⇔	⇔	⇔
	workplaces	⇔	⇔	⇔	⇔	⇔	⇔	⇔
	residences	⇔	⇔	⇔	⇔	⇔	⇔	⇔
QC	retail and recreation	↓	↑	↑	↑	↓	↑	↓
	grocery and pharmacy	↑	⇔	↓	↑	↑	↓	↑
	parks	↓	↓	↑	↑	↑	↓	↓
	transit stations	↑	↓	↑	↑	↓	↑	↓
	workplaces	⇔	↑	↑	↑	↓	↓	↓
	residences	↓	↑	⇔	⇔	⇔	⇔	⇔
SK	retail and recreation	⇔	⇔	⇔	⇔	⇔	⇔	⇔
	grocery and pharmacy	⇔	⇔	⇔	⇔	⇔	⇔	⇔
	parks	↑	⇔	↑	↓	⇔	⇔	⇔
	transit stations	⇔	⇔	⇔	⇔	⇔	⇔	⇔
	workplaces	⇔	⇔	↑	↓	⇔	⇔	⇔
	residences	⇔	⇔	⇔	⇔	⇔	⇔	⇔

Alberta (AB), British Columbia (BC), Manitoba (MB), New Brunswick (NB), Newfoundland and Labrador (NL), Nova Scotia (NS), Ontario (ON), Quebec (QC), Saskatchewan (SK). *T*_1_ (Mar 1, 2020): Entertainment/cultural sector closure. *T*_2_ (Mar 13, 2020): School closure. *T*_3_ (Apr 6, 2020): Mask wearing. *T*_4_ (Apr 21, 2020): Imposed entertainment/cultural sector closure. *T*_5_ (Jul 7, 2020): Imposed entertainment/cultural sector closure. *T*_6_ (Sep 1, 2020): Imposed entertainment/cultural sector closure. *T*_7_ (Nov 2, 2020): Imposed entertainment/cultural sector closure. ⇔ indicates that the range of transmission rate change is between -10% and 10%. ↑ indicates that the range of transmission rate change is between 10% and 100%. ↓ indicates that the variation of transmission rates is between -100% and -10%.

We investigate where the transmission rate (*β*_*j*_(*t*)) is the most important. We calculate the transmission rate (*β*_*j*_(*t*)) of each place in the simulations from February 15 to December 13, 2020 (see Fig 7), and we find that the average transmission rates over the entire simulation period are relatively large in the grocery and pharmacy, and transit stations, which are 0.2584 and 0.2587, respectively, due to the high population density in the grocery and pharmacy, and transit stations. The average transmission rates over the entire simulation period are relatively small in the workplaces and parks, which are 0.2370 and 0.2388, respectively, because most people stay home during the epidemic, thereby reducing the population density of the workplaces. In addition, the average transmission rates of the retail and recreation, and residences during the entire simulation period are 0.2464 and 0.2520, respectively.

### The infection probability in types of different places

**Fig 8:**
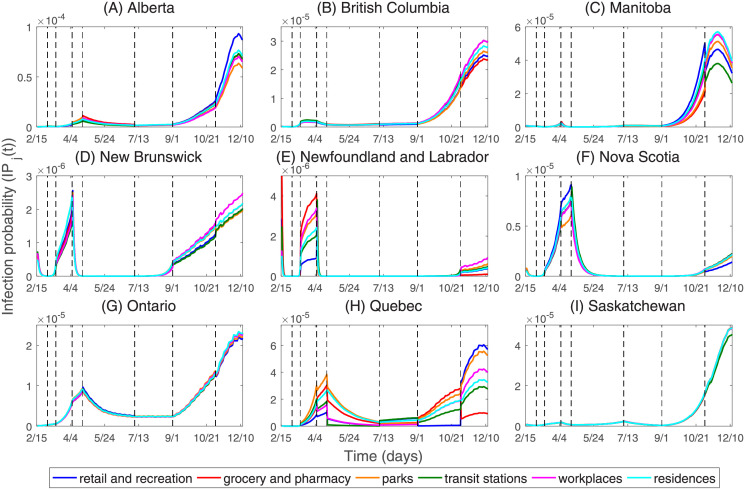
The infection probabilities in retail and recreation, grocery and pharmacy, parks, transit stations, workplaces, and residences in nine Canadian provinces. The black dotted line indicates the time when NPIs are implemented as follows. *T*_1_ (Mar 1, 2020): Entertainment/cultural sector closure. *T*_2_ (Mar 13, 2020): School closure. *T*_3_ (Apr 6, 2020): Mask wearing. *T*_4_ (Apr 21, 2020): Imposed entertainment/cultural sector closure. *T*_5_ (Jul 7, 2020): Imposed entertainment/cultural sector closure. *T*_6_ (Sep 1, 2020): Imposed entertainment/cultural sector closure. *T*_7_ (Nov 2, 2020): Imposed entertainment/cultural sector closure.

**Fig 9:**
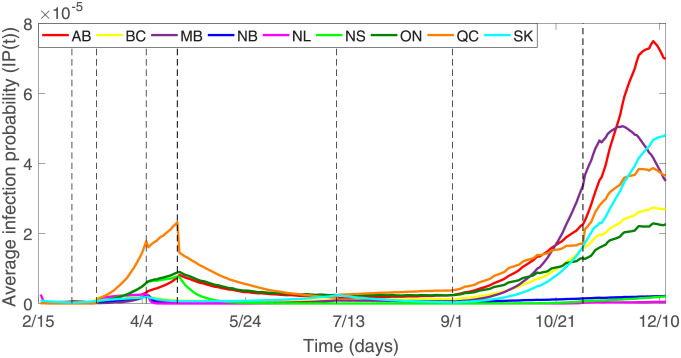
The average infection probability in nine Canadian provinces. The black dotted line indicates the time when NPIs are implemented as follows. *T*_1_ (Mar 1, 2020): Entertainment/cultural sector closure. *T*_2_ (Mar 13, 2020): School closure. *T*_3_ (Apr 6, 2020): Mask wearing. *T*_4_ (Apr 21, 2020): Imposed entertainment/cultural sector closure. *T*_5_ (Jul 7, 2020): Imposed entertainment/cultural sector closure. *T*_6_ (Sep 1, 2020): Imposed entertainment/cultural sector closure. *T*_7_ (Nov 2, 2020): Imposed entertainment/cultural sector closure.

Based on the estimated parameters in Table C.1 of S1 File, and the value of variables *E*, *A*, *I* and *N* obtained by the model, we calculate the probability of susceptible individuals being infected in types of different places in nine Canadian provinces, as shown in Fig 8. We observe that the trend of the daily infection probability is consistent with the number of daily confirmed cases. As of December 13, 2020, in Alberta, British Columbia, Manitoba, New Brunswick, Newfoundland and Labrador, Nova Scotia, Ontario, Quebec, and Saskatchewan, the maximum average infection probabilities in six types of different places are 7.49 × 10^−5^, 2.74 × 10^−5^, 5.06 × 10^−5^, 2.26 × 10^−6^, 2.67 × 10^−6^, 7.69 × 10^−6^, 2.29 × 10^−5^, 3.86 × 10^−5^, and 4.82 × 10^−5^, respectively, which occurs on December 7, December 7, November 22, April 8, April 6, April 21, December 7, December 7, and December 13, respectively. Fig 9 shows that the average infection probability in Quebec is higher than those of the other eight provinces from mid-March 2020 until mid-October 2020. After mid-October 2020, the average infection probability in Manitoba exceeded the average infection probability in Quebec. After November 20, 2020, the average infection probability in Alberta has increased very rapidly and exceeded the average infection probability in Manitoba, and the average infection probability in Alberta has reached the highest value since the onset of the COVID-19 epidemic.

### Uncertainty and sensitivity analysis

**Fig 10:**
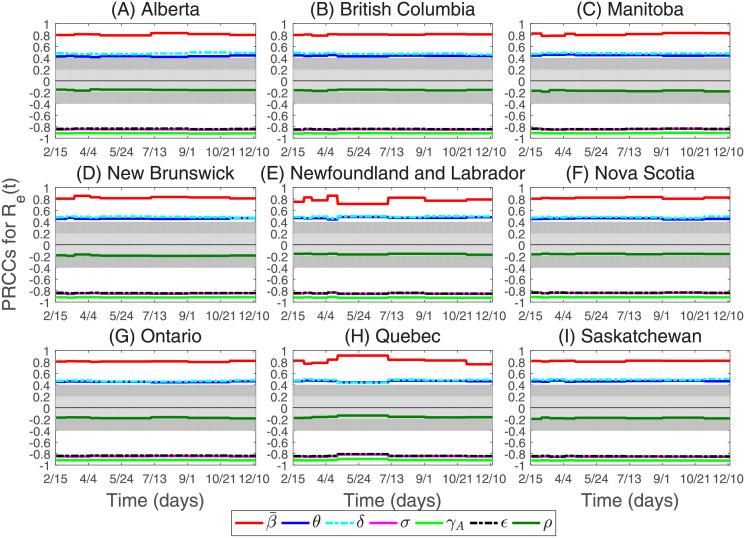
The sensitivity of the parameters varies with the average effective reproduction number *R*_*e*_(*t*). The light gray area represents PRCC values that are not statistically significant. The dark gray areas represent PRCC values that are moderate correlation.

As there are lots of the parameters involved in the model, variations of the parameters may affect the trend of the epidemic greatly. To study the sensitivity of parameters on the model prediction results, we conduct Latin Hypercube Sampling (LHS) and Partial Rank Correlation Coefficients (PRCCs) analysis [36]. The goal is to identify most important parameters affecting the average effective reproduction number *R*_*e*_(*t*). The input parameters are the average transmission rate (i.e. $\bar{\beta }$), the factor for reduced transmission probability of asymptomatic infected individuals (i.e. *θ*), the factor for reduced transmission probability of exposed individuals (i.e. *δ*), the mean length of incubation period (i.e. 1/*σ*), the asymptomatic infectious period (i.e. 1/*γ*_*A*_), the average time to case detection (i.e. 1/*ϵ*) and the proportion of asymptomatic infected individuals (i.e. *ρ*), the output variables is the average effective reproduction number *R*_*e*_(*t*). We choose that all input parameters are normally distributed. We consider absolute values of PRCC greater than 0.4 as indicating the high correlation between input parameters and output variables, values between 0.2 and 0.4 as moderate correlations, and values between 0 and 0.2 are not significantly different from zero [37, 38]. The results of the sensitivity analysis of parameters are shown in Fig 10.

Fig 10 illustrates the PRCCs for the dependence of the average effective reproduction number in nine Canadian provinces on each parameter form February 15, 2020 to December 13, 2020. We found that the parameters $\bar{\beta }$, *θ* and *δ* are highly positively correlated with the average effective reproduction number, and the correlation coefficient between $\bar{\beta }$ and the average effective reproduction number is the highest, which indicates that reducing parameter $\bar{\beta }$ has a greater impact on the COVID-19 epidemic than parameters *θ* and *δ*. The parameters *σ*, *γ*_*A*_ and *ϵ* are highly negatively correlated with the average effective reproduction number, and the correlation coefficient between *γ*_*A*_ and the average effective reproduction number is the lowest, which indicates that the early detection of asymptomatic infectious cases has a greater impact on the COVID-19 epidemic than parameters *σ* and *ϵ*.

## Discussion

The government of Canada has initiated a series of NPIs to contain the spread of the COVID-19 epidemic [6]. Public heath policies vary in space and time, resulting in varied patterns of movement and behavioural changes throughout the country. Due to the changes in movement patterns and behavioural changes of humans, the severity of the COVID-19 outbreak in Canada varies from place to place.

To understand how human mobility, the transmission rate, and infection probability affect the spread of the COVID-19 epidemic, we developed a meta-population model for the transmission dynamics of COVID-19, where the human mobility is characterized by a dynamic network. The model takes into account the heterogeneity between different provinces in Canada, such as the timing of implementing NPIs, the human mobility in retail and recreation, grocery and pharmacy, parks, transit stations, workplaces, and residences due to work, and recreation.

We only analyzed the epidemic in nine Canadian provinces from February 15, 2020 to December 13, 2020, including Alberta (AB), British Columbia (BC), Manitoba (MB), New Brunswick (NB), Newfoundland and Labrador (NL), Nova Scotia (NS), Ontario (ON), Quebec (QC), and Saskatchewan (SK), since the COVID-19 epidemic in other Canadian provinces is not serious due to low population densities.

As is known, the effect of human mobility on the spatial and temporal spread of COVID-19 is delayed a few weeks due to the incubation period of COVID-19 infection and reporting delay [39–41]. To determine which activity is most closely related to the dynamics of COVID-19, we used the cross-correlation analysis [14] to identify the places where the human mobility data are statistically significant and correlated with the weekly number of confirmed cases from February 15 to December 13, 2020. We found that activities in locations around the residences can reduce the severity of COVID-19, and when the time-lag is about 16 weeks, the positive correlation is the highest between the mobility data of parks and the weekly number of confirmed COVID-19 cases.

Our model has incorporated many key factors responsible for the temporal and spatial spread of the COVID-19 epidemic, such as the timing of implementing NPIs and the human mobility. Since intra-province travel contributes more to the increase of cumulative number of cases than inter-province travel [9, 11] due to substantially reduced long-distance travels, our model only considered intra-province human mobility. We found that the average effective reproduction numbers in nine Canadian provinces are all greater than one from February 15, 2020 to December 13, 2020. NPIs have little impact on the epidemics in Ontario and Saskatchewan. After November 20, 2020, the average infection probability of Alberta has reached the highest since the COVID-19 epidemic in Canada. We carried out the sensitivity analysis of the average effective reproduction number with respect to the model parameters. The result identified six key parameters: the average transmission rate (i.e. $\bar{\beta }$), the factor for reduced transmission probability of asymptomatic infected individuals (i.e. *θ*), the factor for reduced transmission probability of exposed individuals (i.e. *δ*), the mean length of incubation period (i.e. 1/*σ*), the asymptomatic infectious period (i.e. 1/*γ*_*A*_), the average time to case detection (i.e. 1/*ϵ*), play an important role in the size of the average effective reproduction number. This suggested that the transmission rate, the mean length of incubation period, the asymptomatic infectious period, and the average time to case detection play an important role during the COVID-19 epidemic. Our findings provide insights that help support the planning of emergency management and decision-making for public health authorities. However, our study still has several limitations. First, since the numbers of asymptomatic infected individuals and recovered individuals are not publicly available yet, our simulations only used incidence data and death cases. Second, behavior and personal risk management such as masks, no indoor gatherings, social distancing, compliance with quarantine measures are intertwined with movement and hard to disentangle from the movement data. In fact, we are likely seeing the results not of movement alone but of a combination of movement, NPI, and individual behavior change as a result of public health orders but also perceived risk and information from surrounding areas. Third, we did not consider the effectiveness of contact tracing and the speed of tracing, and the human mobility between provinces and the imported cases from overseas and the role of environmental factors, which will be studied in future work when such data become available.

## Supporting information

S1 File Data collection and analysis, the details of the cross-correlation function, the mean values and standard deviation of parameters and initial for Models (1) and (2). (ZIP)
