# Supplementary material for: Evaluating the impacts of non-pharmaceutical interventions on the transmission dynamics of COVID-19 in Canada based on mobile network
Source: PLoS One. 2021 Dec 29;16(12):e0261424. doi: 10.1371/journal.pone.0261424 (PMC8716046; doi:10.1371/journal.pone.0261424)
Supplement: S1 File — (ZIP) [file pone.0261424.s001.zip › SI17886.pdf]

Supporting Information (SI): Evaluating the impacts of non-pharmaceutical interventions on the transmission dynamics of COVID-19 in Canada based on mobile network

Ling Xue[1]*, Shuanglin Jing[1*], Hao Wang[2,†]

[1]College of Mathematical Sciences, Harbin Engineering University, Harbin, Heilongjiang, 150001, China

[2]Department of Mathematical and Statistical Sciences, University of Alberta, Edmonton, Alberta, AB T6G 2R3, Canada

E-mail: hao8@ualberta.ca

Appendix

A Data collection and analysis

We obtained the number of COVID-19 cases in nine Canadian provinces from Johns Hopkins University Center for Systems Science and Engineering (see Fig A.1). The mobility data is obtained from Google COVID-19 Community Mobility Reports (CMR) at `https://google.com/covid19/mobility` as is plotted in Fig A.2. The cross-correlation analysis between Google Community Mobility Reports data and the weekly number of confirmed death cases are shown in Fig A.3.

*Contributed equally.

†Corresponding author.

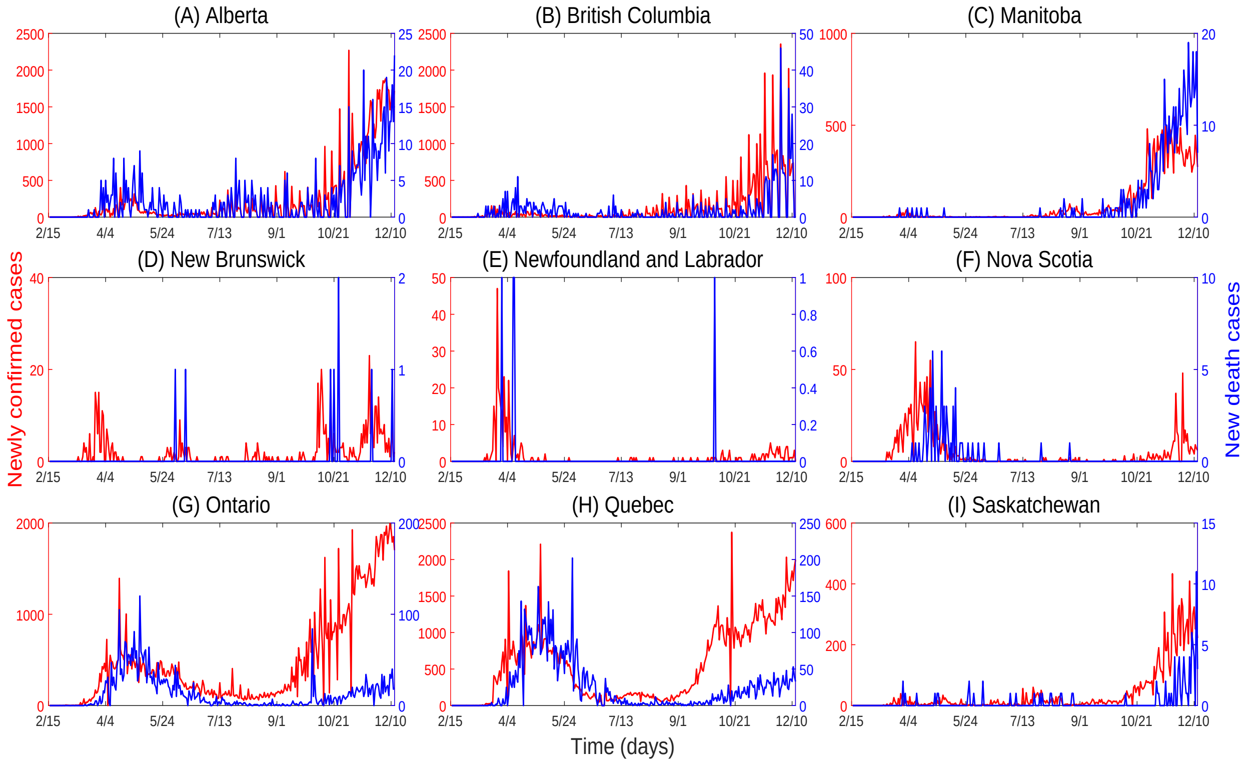

Fig A.1: **Daily number of confirmed cases and daily number of confirmed deaths in Canada from February 15, 2020 to December 13, 2020.**

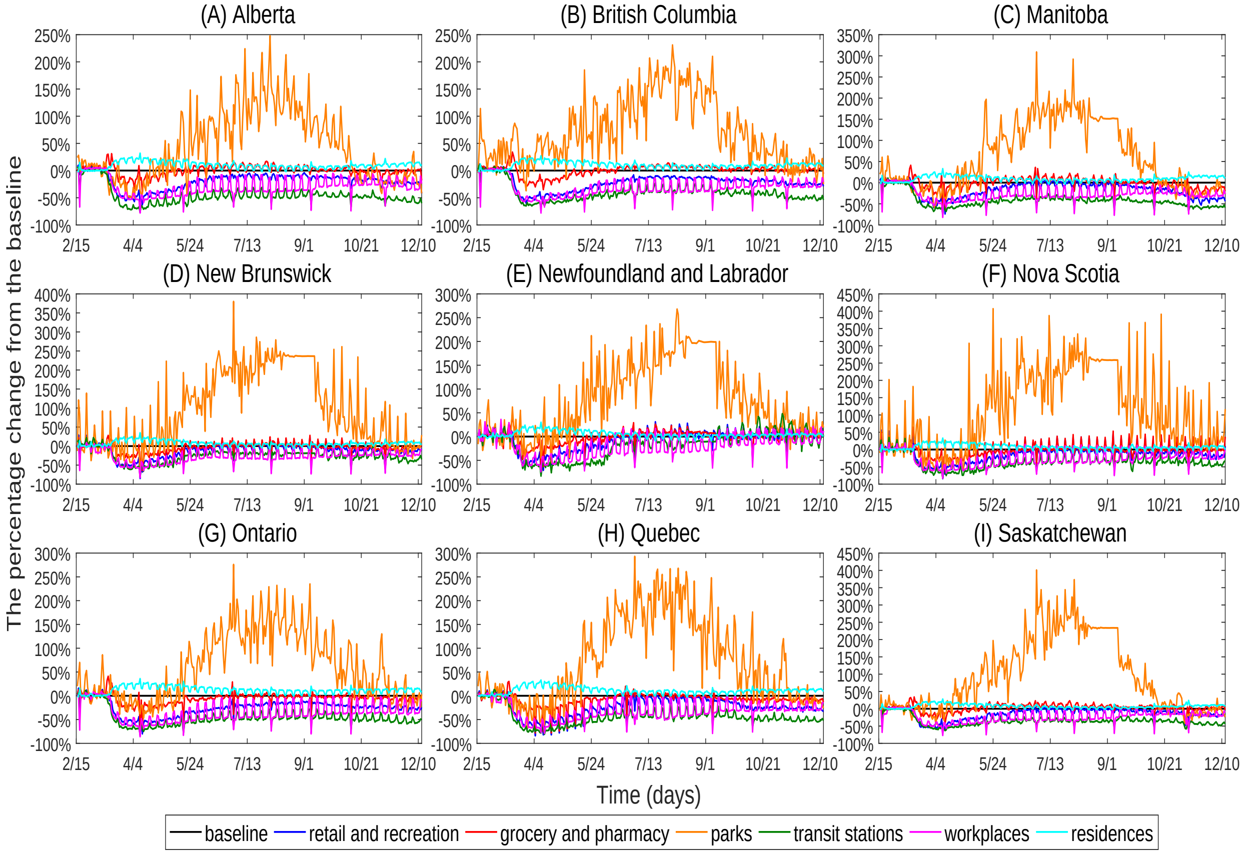

Fig A.2: Mobility data retrieved from Google Community Mobility Reports in Canada between February 15, 2020 and December 13, 2020.

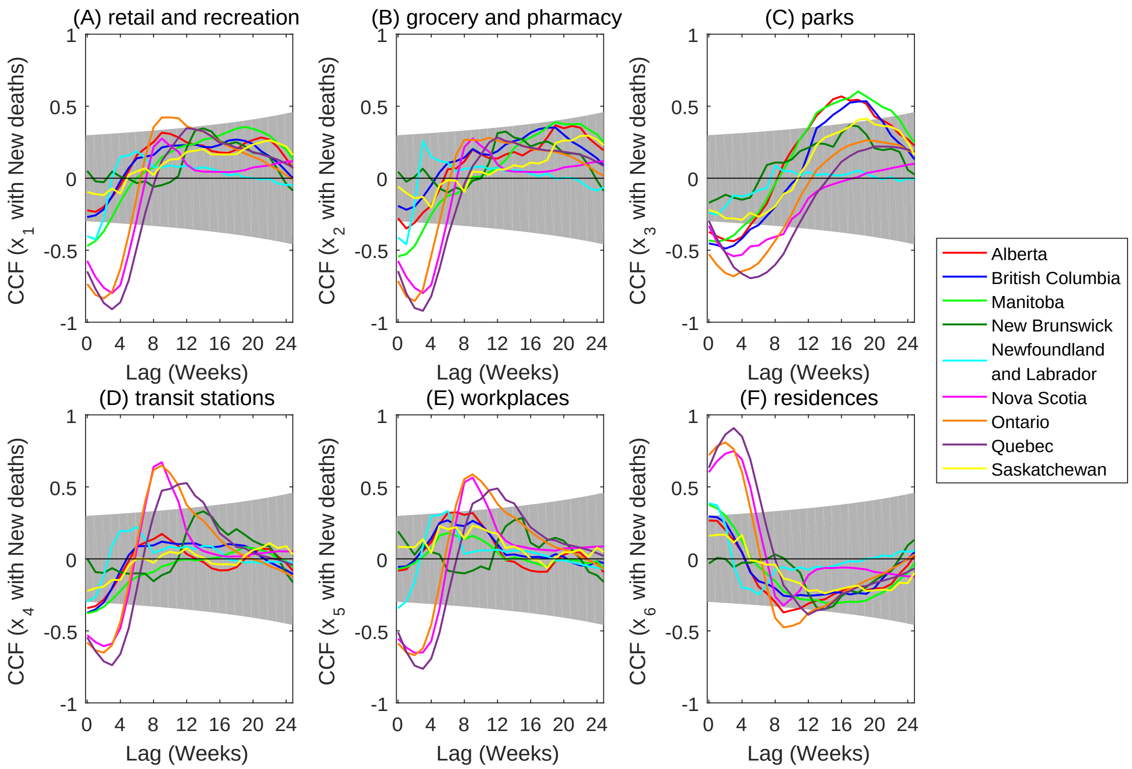

Fig A.3: **Correlations between Google Community Mobility Reports data and the weekly number of confirmed deaths whin different lags (in weeks) for each region.** The variables x_1(A), x_2(B), x_3(C), x_4(D), x_5(E), and x_6(F) denote retail and recreation, grocery and pharmacy, parks, transit stations, workplaces, and residences. The gray area represents the 95% confidence intervals.

B The details of the cross-correlation function

The cross-correlation function (CCF) describes the correlation between two random processes x_t and y_t ($t = 0, \pm 1, \pm 2, \cdots$) at any two different times. We assume that x_t and y_t are both one-dimensional stationary processes, and the cross-covariance function $\text{Cov}(x_t, y_s)$ between x_t and y_s is only a function of time difference $(s - t)$, then x_t and y_t are joint stationary [1]. Therefore, we can get the cross covariance function of x_t and y_t as follows

$$\gamma_{xy}(k) = E[x_t - \mu_x][y_{t+k} - \mu_y], k = 0, \pm 1, \pm 2, \cdots, \tag{B.1}$$

where $\mu_x = E(x_t)$ and $\mu_y = E(y_t)$. After normalizing Eq.(B.1), we get the cross-correlation function as follows

$$\rho_{xy}(k) = \frac{\gamma_{xy}(k)}{\sigma_x \sigma_y}, k = 0, \pm 1, \pm 2, \cdots,$$

where σ_x and σ_y are the standard deviations of two random processes x_t and y_t.

In general, we use the sample cross-correlation function to estimate the overall cross-correlation function as follows

$$\hat{\rho}_{xy}(k) = \frac{\hat{\gamma}_{xy}(k)}{S_x S_y}, k = 0, \pm 1, \pm 2, \cdots,$$

where S_x and S_y are the standard deviation of samples of two random processes x_t and y_t, and $S_x = \sqrt{\hat{\gamma}_{xx}(0)}$, $S_y = \sqrt{\hat{\gamma}_{yy}(0)}$ and

$$\hat{\gamma}_{xy}(k) = \begin{cases} \frac{1}{n}\sum_{t=1}^{n-k}(x_t - \overline{x})(y_{t+k} - \overline{y}), & k \geq 0, \\ \frac{1}{n}\sum_{t=1-k}^{n}(x_t - \overline{x})(y_{t+k} - \overline{y}), & k < 0, \end{cases}$$

where $\overline{x}$ and $\overline{y}$ are the mean values of the samples of two random processes x_t and y_t. If two random processes are independent or uncorrelated, then the cross-correlation function value for all lags is zero. If the cross-correlation function $\hat{\rho}_{xy}(k)$ reaches a local maximum at the lag $k = d$, then it means that the lag between two random processes is d.

In order to test if a CCF $\rho_{xy}(k)$ value is significantly different from zero, Bartlett proved that the CCF $\hat{\rho}_{xy}(k)$ is approximately normally distributed with zero mean and a variance of $1/(n-k)$ [2]. Thus, we obtain the following statistic

$$U = \frac{\hat{\rho}_{xy}(k)}{\sqrt{\frac{1}{n-k}}} \sim N(0, 1),$$

where n is the number of observed samples. The 95% confidence interval can be expressed as

$$\left[0 - 1.96\sqrt{\frac{1}{n-k}}, 0 + 1.96\sqrt{\frac{1}{n-k}}\right].$$

We used this statistic to calculate a 95% critical region which would encompass the estimated cross correlation of uncorrelated processes over 95% of the lags. If the estimated CCF $\hat{\rho}_{xy}(k)$ markedly differs from the critical region at a lag k, then we interpret the value as being significantly different from zero (i.e. $\rho_{xy}(k) \neq 0$), otherwise $\rho_{xy}(k) = 0$.

C The mean values and standard deviation of parameters and initial values for Models (1) and (2).

Table C.1: The mean values of parameters and initial values for Models (1) and (2).

Parameters	AB	BC	MB	NB	NL	NS	ON	QC	SK
E(0)	4.1993	4.5305	5.4237	3.3944	7.9191	4.2744	5.0859	7.3016	5.0970
P_{HR}	0.001344	0.001485	0.004600	0.002640	0.013124	0.007708	0.000122	0.005676	0.002538
a_{11}	0.2431	0.2419	0.2758	0.2543	0.3520	0.2984	0.2520	0.2025	0.2623
a_{12}	0.2475	0.2236	0.2579	0.2887	0.1061	0.2213	0.2565	0.3882	0.2545
a_{13}	0.1772	0.2630	0.3243	0.2165	0.1091	0.2960	0.2486	0.1299	0.2514
a_{14}	0.2095	0.2156	0.1780	0.2619	0.3572	0.2907	0.2498	0.1002	0.2568
a_{15}	0.2117	0.2242	0.2489	0.3091	0.3519	0.2592	0.2664	0.0526	0.2468
a_{16}	0.2263	0.1977	0.2533	0.2105	0.2218	0.2105	0.2496	0.3490	0.2435
a_{17}	0.2844	0.2179	0.3245	0.2106	0.4963	0.2690	0.2482	0.0080	0.2495
a_{18}	0.3173	0.2255	0.2097	0.2315	0.2287	0.1653	0.2408	0.4532	0.2553
a_{21}	0.2383	0.2727	0.2125	0.2399	0.6230	0.2271	0.2602	0.2812	0.2393
a_{22}	0.2693	0.2162	0.2437	0.2665	0.4025	0.1830	0.2273	0.2094	0.2549
a_{23}	0.2514	0.2835	0.2512	0.2004	0.4601	0.2304	0.2388	0.2285	0.2469
a_{24}	0.2466	0.2578	0.3178	0.2022	0.1159	0.2547	0.2548	0.2964	0.2401
a_{25}	0.3527	0.2961	0.2899	0.2052	0.6365	0.2628	0.2374	0.1848	0.2496
a_{26}	0.2008	0.2753	0.2806	0.2466	0.3283	0.2583	0.2413	0.1524	0.2432
a_{27}	0.2649	0.2521	0.1331	0.2607	0.2076	0.2157	0.2479	0.3774	0.2389
a_{28}	0.2474	0.2135	0.2495	0.2481	0.0472	0.2475	0.2531	0.0738	0.2561
a_{31}	0.2803	0.2285	0.2060	0.2609	0.2581	0.3256	0.2423	0.1136	0.2672
a_{32}	0.2106	0.2737	0.2889	0.2187	0.0853	0.2547	0.2518	0.3005	0.2352
a_{33}	0.2531	0.2952	0.3050	0.2535	0.3362	0.2336	0.2621	0.4672	0.2478
a_{34}	0.3249	0.2752	0.2173	0.2549	0.0591	0.1914	0.2610	0.3746	0.2197
a_{35}	0.2652	0.2239	0.2930	0.2651	0.0554	0.2028	0.2413	0.2789	0.2612
a_{36}	0.2312	0.2668	0.1618	0.2721	0.1114	0.2227	0.2594	0.1367	0.2612
a_{37}	0.2147	0.2127	0.1492	0.2459	0.0908	0.2280	0.2689	0.3221	0.2473
a_{38}	0.2154	0.2377	0.2309	0.2267	0.3461	0.2354	0.2462	0.4173	0.2540
a_{41}	0.2853	0.2942	0.2109	0.2966	0.3072	0.2211	0.2570	0.3828	0.2496
a_{42}	0.3401	0.2358	0.3685	0.1988	0.1871	0.2175	0.2458	0.1608	0.2636
a_{43}	0.2588	0.3059	0.2442	0.1753	0.2227	0.2267	0.2555	0.3121	0.2502
a_{44}	0.1959	0.2803	0.2608	0.2108	0.4032	0.2395	0.2356	0.1796	0.2544
a_{45}	0.1904	0.2409	0.2620	0.2838	0.5322	0.2793	0.2540	0.0091	0.2566
a_{46}	0.2231	0.2671	0.2735	0.2371	0.2865	0.1957	0.2502	0.3674	0.2344
a_{47}	0.2503	0.2656	0.2348	0.1994	0.6068	0.2481	0.2499	0.1678	0.2447
a_{48}	0.2490	0.2713	0.1714	0.2312	0.2675	0.2660	0.2558	0.2201	0.2374
a_{51}	0.2250	0.2783	0.2246	0.2758	0.1543	0.2045	0.2541	0.3016	0.2542
a_{52}	0.2153	0.2493	0.2620	0.3038	0.1573	0.2888	0.2586	0.2913	0.2526
a_{53}	0.2292	0.2177	0.2675	0.1849	0.3619	0.2412	0.2411	0.2555	0.2603
a_{54}	0.2578	0.2483	0.2503	0.2474	0.1300	0.2377	0.2373	0.1548	0.2311
a_{55}	0.2845	0.2345	0.2265	0.2190	0.3614	0.1807	0.2485	0.0563	0.2542
a_{56}	0.2249	0.2684	0.2387	0.1710	0.1926	0.2324	0.2451	0.0700	0.2580
a_{57}	0.2040	0.2882	0.2056	0.2661	0.0959	0.2840	0.2614	0.2546	0.2517
a_{58}	0.2381	0.2719	0.2501	0.2835	0.5360	0.2557	0.2522	0.3184	0.2543
a_{61}	0.2538	0.2340	0.2418	0.1948	0.1213	0.2047	0.2570	0.1638	0.2376
a_{62}	0.2432	0.2821	0.2264	0.3149	0.4563	0.3027	0.2511	0.3925	0.2571
a_{63}	0.2627	0.2509	0.2680	0.2571	0.2543	0.2608	0.2622	0.2629	0.2540
a_{64}	0.2578	0.2455	0.2225	0.2015	0.1508	0.2550	0.2561	0.2583	0.2439
a_{65}	0.2474	0.2505	0.2532	0.2458	0.1831	0.2403	0.2497	0.2550	0.2502
a_{66}	0.2516	0.2483	0.2481	0.2634	0.3240	0.2469	0.2504	0.2587	0.2428
a_{67}	0.2563	0.2535	0.2584	0.2497	0.2928	0.2581	0.2543	0.2577	0.2551
a_{68}	0.2607	0.2540	0.2569	0.2493	0.2399	0.2523	0.2562	0.2590	0.2552

Alberta (AB), British Columbia (BC), Manitoba (MB), New Brunswick (NB), Newfoundland and Labrador (NL), Nova Scotia (NS), Ontario (ON), Quebec (QC), Saskatchewan (SK).

Table C.2: The standard deviation of parameters and initial values for Models (1) and (2).

Parameters	AB	BC	MB	NB	NL	NS	ON	QC	SK
E(0)	5.2258E-01	1.4118E-01	2.2244E-01	3.3914E-01	1.3968E-00	5.9641E-02	4.0908E-02	9.8925E-02	1.2058E-01
P_{HR}	8.3151E-04	9.8154E-04	1.4125E-03	1.2989E-04	3.1615E-03	4.0088E-04	8.9149E-05	1.0759E-04	8.2871E-04
a_{11}	1.3458E-02	1.3640E-02	6.8478E-03	2.3783E-03	7.2029E-02	3.0322E-03	9.4682E-04	9.9461E-03	1.0479E-02
a_{12}	9.5728E-03	4.8681E-03	5.5847E-03	1.0870E-02	6.1367E-02	3.1574E-03	5.0445E-04	5.0172E-03	6.7238E-03
a_{13}	7.8493E-02	2.4269E-02	1.8501E-02	1.5305E-02	4.8255E-02	3.6812E-03	1.2074E-03	5.1418E-03	4.7007E-03
a_{14}	2.5597E-02	1.7089E-02	2.0310E-02	1.6637E-03	7.1389E-02	2.3028E-03	4.5748E-04	8.7484E-03	3.2753E-03
a_{15}	3.2637E-02	1.0893E-02	5.8168E-03	1.4239E-02	6.4100E-02	2.6876E-03	1.3481E-03	1.0675E-02	8.1192E-03
a_{16}	4.4979E-02	9.4095E-03	2.2848E-03	6.1356E-03	2.8827E-02	2.1862E-03	3.5214E-04	4.1859E-03	4.6343E-03
a_{17}	3.4606E-02	5.6252E-03	1.9081E-02	1.6637E-02	4.8027E-02	1.8895E-03	1.6282E-03	8.9132E-03	1.0041E-02
a_{18}	5.1172E-02	7.3240E-03	1.5094E-02	5.0684E-03	3.2169E-02	4.5254E-03	5.2214E-04	9.6655E-03	8.2056E-03
a_{21}	1.8222E-02	9.8808E-03	1.3066E-02	3.7694E-03	1.3076E-01	1.8862E-03	3.4925E-04	5.9608E-03	7.2827E-03
a_{22}	3.4162E-02	8.4390E-03	3.5288E-03	3.1892E-03	2.5539E-02	4.6441E-03	2.5216E-03	1.0555E-02	1.2212E-02
a_{23}	9.8870E-03	5.9513E-03	4.4553E-03	7.9932E-03	6.8339E-02	4.2263E-03	1.7520E-03	1.2428E-03	1.0434E-02
a_{24}	8.6251E-03	6.2460E-03	2.1080E-02	8.8341E-03	4.5261E-02	1.2830E-03	5.6504E-04	2.6563E-03	3.4495E-03
a_{25}	9.4856E-02	1.0748E-02	6.9795E-03	9.3353E-03	6.7403E-02	1.5921E-03	7.6387E-04	4.0413E-03	2.9777E-03
a_{26}	4.9631E-02	8.2633E-03	1.0739E-02	9.8345E-03	1.1993E-01	1.3633E-03	3.9794E-04	3.2599E-03	2.7929E-03
a_{27}	1.0993E-02	1.4850E-03	2.8224E-02	2.6771E-03	6.3510E-02	1.7196E-03	1.4758E-03	1.0456E-02	4.6459E-03
a_{28}	1.6189E-02	5.4591E-03	1.9811E-03	7.8158E-03	4.1166E-02	3.0025E-03	4.1155E-04	5.7668E-03	4.3782E-03
a_{31}	2.5495E-02	1.6049E-02	1.5998E-02	1.2956E-02	5.6852E-02	5.4665E-03	5.7425E-04	6.3894E-03	7.8584E-03
a_{32}	4.3528E-02	1.1150E-02	4.7053E-03	2.6403E-03	4.1426E-02	1.9575E-03	8.2277E-04	1.7897E-03	1.5161E-02
a_{33}	6.1539E-03	1.9345E-02	1.4822E-02	7.5635E-03	2.7025E-02	2.1763E-03	9.0622E-04	5.6827E-03	8.8743E-03
a_{34}	7.3417E-02	9.0614E-03	5.6487E-03	3.6368E-03	3.9774E-02	4.5111E-03	1.8879E-03	3.7887E-03	8.1312E-03
a_{35}	8.3200E-03	1.8154E-02	1.5157E-02	3.4655E-03	3.4096E-02	3.3222E-03	2.0722E-04	1.0655E-02	2.8771E-03
a_{36}	1.8832E-02	1.2169E-02	2.3354E-02	8.5634E-03	1.0089E-01	2.5515E-03	1.3665E-03	1.1694E-02	4.9652E-03
a_{37}	3.7870E-02	9.5117E-03	2.5543E-02	5.0862E-03	4.4528E-02	1.9620E-03	3.2243E-04	3.4558E-03	5.7702E-03
a_{38}	2.6663E-02	4.6671E-03	6.0159E-03	6.9010E-03	2.5877E-02	2.3086E-03	4.4976E-04	4.7582E-03	3.1187E-03
a_{41}	5.0225E-02	1.1217E-02	1.0078E-02	4.2188E-03	8.2971E-02	2.2808E-03	1.2585E-03	1.0864E-02	3.8184E-03
a_{42}	7.9031E-02	1.4374E-02	2.4355E-02	7.5195E-03	7.2301E-02	1.9700E-03	4.7822E-04	2.5887E-03	4.0667E-03
a_{43}	4.1486E-03	1.0009E-02	5.5680E-03	2.6295E-02	7.8726E-02	2.2593E-03	1.6017E-03	2.1895E-03	4.2938E-03
a_{44}	4.0915E-02	2.0349E-02	3.8921E-03	1.3017E-02	3.8110E-02	2.2652E-03	9.5124E-04	3.5748E-03	1.7932E-03
a_{45}	6.6016E-02	6.1292E-03	3.3995E-03	7.8460E-03	1.1550E-01	2.7520E-03	2.2968E-03	7.0096E-03	4.0865E-03
a_{46}	4.6869E-02	1.4733E-02	6.6870E-03	6.1026E-03	5.5108E-02	2.9128E-03	5.0745E-04	5.7992E-03	9.2711E-03
a_{47}	5.2093E-03	1.4061E-02	5.8894E-03	1.6518E-02	3.1991E-02	1.4028E-03	1.9994E-03	2.6508E-03	7.5511E-03
a_{48}	4.7433E-03	7.5879E-03	1.6619E-02	3.7439E-03	3.1754E-02	6.4893E-04	4.1220E-04	3.2061E-03	3.4694E-03
a_{51}	1.9527E-02	1.4707E-02	1.0029E-02	1.0064E-02	3.6628E-02	2.4599E-03	2.1449E-04	1.9374E-03	3.9321E-03
a_{52}	7.5071E-03	3.4669E-02	7.6428E-03	1.4546E-02	7.0566E-02	1.7644E-03	1.8669E-03	3.6491E-03	1.8809E-03
a_{53}	1.3945E-02	9.8764E-03	5.7883E-03	1.4289E-02	2.3187E-02	2.2116E-03	1.0348E-03	6.8574E-03	2.9424E-03
a_{54}	3.7239E-03	3.1914E-03	4.5642E-03	1.3096E-02	6.4117E-02	1.4824E-03	9.4325E-04	3.1042E-03	7.8194E-03
a_{55}	1.8409E-02	5.7448E-03	8.5020E-03	7.8355E-03	8.6090E-02	4.5745E-03	7.6803E-04	5.8489E-03	2.7373E-03
a_{56}	2.0737E-02	7.2251E-03	4.7176E-03	1.9498E-02	7.9228E-02	2.9654E-03	4.3662E-04	1.0330E-02	7.2313E-03
a_{57}	5.5009E-02	1.2637E-02	1.2660E-02	1.3026E-02	8.6485E-02	2.2552E-03	2.9520E-03	4.6670E-03	4.9548E-03
a_{58}	2.1346E-02	1.7655E-02	5.0279E-03	2.8560E-03	8.8834E-02	1.1739E-03	5.8973E-04	2.1187E-03	3.5807E-03
a_{61}	5.7104E-03	9.5419E-03	3.9203E-03	8.9522E-03	7.1505E-03	1.6597E-03	1.1218E-03	2.7487E-03	4.2251E-03
a_{62}	8.3006E-03	1.2135E-02	8.4033E-03	1.4925E-02	1.6224E-02	2.8137E-03	4.2204E-04	4.0504E-03	5.4213E-03
a_{63}	3.3754E-03	1.1521E-03	7.7950E-03	5.8745E-04	1.0902E-03	2.2668E-04	7.4821E-04	9.3751E-04	2.3783E-03
a_{64}	1.4365E-03	2.3686E-03	8.6640E-03	3.8509E-03	1.7125E-02	1.5124E-04	4.6883E-04	4.2450E-04	5.0908E-03
a_{65}	5.9405E-04	6.9479E-04	1.7304E-03	2.8408E-03	2.1253E-02	3.1664E-04	1.2789E-04	2.0923E-04	8.0528E-04
a_{66}	1.0877E-03	5.1897E-04	6.2638E-04	2.5020E-03	3.0464E-02	1.2678E-03	1.3388E-04	2.9587E-04	5.1700E-04
a_{67}	1.4668E-03	3.9810E-04	4.9924E-04	6.3056E-04	2.3220E-02	9.8815E-04	6.8096E-05	2.0284E-04	2.9532E-04
a_{68}	5.0585E-04	4.8353E-04	1.2004E-04	3.2854E-04	3.3785E-03	2.1857E-04	8.8959E-05	2.5551E-04	1.3894E-04

Alberta (AB), British Columbia (BC), Manitoba (MB), New Brunswick (NB), Newfoundland and Labrador (NL), Nova Scotia (NS), Ontario (ON), Quebec (QC), Saskatchewan (SK).

References

[1] El-Gohary M, McNames J. Establishing causality with whitened cross-correlation analysis. IEEE Transactions on Biomedical Engineering. 2007;54(12):2214–2222.

[2] Bartlett M. Stochastic Processes. Cambridge University Press, Cambridge; 1955.
